# Supplementary material for: Association of Dairy Product Consumption with Metabolic and Inflammatory Biomarkers in Adolescents: A Cross-Sectional Analysis from the LabMed Study
Source: Nutrients. 2019 Sep 21;11(10):2268. doi: 10.3390/nu11102268 (PMC6835390; doi:10.3390/nu11102268)
Supplement: Supplementary file 1 [file nutrients-11-02268-s001.pdf]

Table S1. Association of cheese intake with C-reactive protein and interleukin-6 concentrations.

Tertiles of cheese intake	C-reactive protein				Interleukin-6			
	Model 1		Model 2		Model 1		Model 2	
	AMR (95% CI)	*P*-value	AMR (95% CI)	*P*-value	AMR (95% CI)	*P*-value	AMR (95% CI)	*P*-value
T1	Reference		Reference		Reference		Reference	
T2	1.26 (0.94; 1.71)	0.124	1.10 (0.82; 1.48)	0.510	0.99 (0.83; 1.18)	0.898	1.02 (0.85; 1.23)	0.801
T3	0.99 (0.71; 1.39)	0.974	0.98 (0.70; 1.38)	0.912	0.94 (0.78; 1.14)	0.541	1.00 (0.81; 1.22)	0.965

AMR, arithmetic mean ratio; T, tertile. Cheese: T1, ≤ 4.3 g/day; T2, 4.3–12.9 g/day; T3, ≥ 12.9 g/day. Model 1: Unadjusted model; Model 2: Adjusted for age, gender, weight status, pubertal stage A and B, total energy intake, mean adequacy ratio, socioeconomic status and cardiorespiratory fitness (VO_2max).

Table S2. Association of total dairy product, milk, yogurt and cheese intake with adiponectin and leptin concentrations.

	Adiponectin				Leptin			
	Model 1		Model 2		Model 1		Model 2	
	AMR (95% CI)	*P*-value	AMR (95% CI)	*P*-value	AMR (95% CI)	*P*-value	AMR (95% CI)	*P*-value
**Tertiles of total dairy products intake**								
T1	Reference		Reference		Reference		Reference	
T2	1.00 (0.90; 1.11)	0.985	1.03 (0.93; 1.13)	0.555	0.92 (0.71; 1.20)	0.555	1.00 (0.82; 1.22)	0.972
T3	0.96 (0.86; 1.07)	0.449	0.96 (0.85; 1.08)	0.467	0.93 (0.71; 1.21)	0.586	1.03 (0.83; 1.27)	0.813
**Tertiles of milk intake**								
T1	Reference		Reference		Reference		Reference	
T2	0.92 (0.82; 1.03)	0.142	0.98 (0.88; 1.10)	0.818	1.03 (0.86; 1.22)	0.760	1.01 (0.83; 1.25)	0.888
T3	0.94 (0.84; 1.06)	0.351	0.98 (0.86; 1.11)	0.715	1.03 (0.86; 1.24)	0.724	1.10 (0.88; 1.39)	0.397
**Tertiles of yogurt intake**								
T1	Reference				Reference		Reference	
T2	0.97 (0.88; 1.08)	0.613	0.98 (0.89; 1.09)	0.694	1.21 (0.95; 1.55)	0.124	0.94 (0.79; 1.12)	0.506
T3	0.91 (0.78; 1.07)	0.243	0.89 (0.76; 1.04)	0.131	1.32 (0.90;1.94)	0.161	1.20 (0.90; 1.60)	0.213
**Tertiles of cheese intake**								
T1	Reference		Reference		Reference		Reference	
T2	0.95 (0.86; 1.05)	0.333	0.98 (0.89; 1.08)	0.685	0.83 (0.65; 1.08)	0.165	0.95 (0.78; 1.14)	0.569
T3	0.97 (0.86; 1.09)	0.590	1.00 (0.89; 1.13)	0.943	0.94 (0.71; 1.25)	0.656	0.98 (0.79; 1.23)	0.885

Total dairy products: T1, ≤ 266.1 g/day; T2, 266.1–506.9 g/day; T3, ≥ 506.9 g/day; Milk: T1, ≤ 192.5 g/day; T2, 192.5–245.0 g/day; T3, ≥ 245.0 g/day; Yogurt: T1, ≤ 53.6 g/day; T2, 53.6–125.0 g/day; T3, ≥ 125.0 g/day; Cheese: T1, ≤ 4.3 g/day; T2, 4.3–12.9 g/day; T3, ≥ 12.9 g/day. Model 1: Unadjusted model; Model 2: Adjusted for age, gender, weight status, pubertal stage A and B, total energy intake, mean adequacy ratio, socioeconomic status and cardiorespiratory fitness (VO2max).
